# High-Accuracy Surface Topography Manufacturing for Continuous Phase Plates Using an Atmospheric Pressure Plasma Jet

**DOI:** 10.3390/mi12060683

**Published:** 2021-06-10

**Authors:** Huiliang Jin, Caixue Tang, Haibo Li, Yuanhang Zhang, Yaguo Li

**Affiliations:** 1 Research Center of Laser Fusion, China Academy of Engineering Physics, Mianyang 621900, China; ispwr@foxmail.com (C.T.); feel612@163.com (H.L.); yuanhang_zhang@126.com (Y.Z.); 2 Chengdu Fine Optical Engineering Research Center, Chengdu 610041, China

**Keywords:** atmospheric pressure plasma jet, continuous phase plate, surface topography, high accuracy and efficiency

## Abstract

The continuous phase plate (CPP) is the vital diffractive optical element involved in laser beam shaping and smoothing in high-power laser systems. The high gradients, small spatial periods, and complex features make it difficult to achieve high accuracy when manufacturing such systems. A high-accuracy and high-efficiency surface topography manufacturing method for CPP is presented in this paper. The atmospheric pressure plasma jet (APPJ) system is presented and the removal characteristics are studied to obtain the optimal processing parameters. An optimized iterative algorithm based on the dwell point matrix and a fast Fourier transform (FFT) is proposed to improve the accuracy and efficiency in the dwell time calculation process. A 120 mm × 120 mm CPP surface topography with a 1326.2 nm peak-to-valley (PV) value is fabricated with four iteration steps after approximately 1.6 h of plasma processing. The residual figure error between the prescribed surface topography and plasma-processed surface topography is 28.08 nm root mean square (RMS). The far-field distribution characteristic of the plasma-fabricated surface is analyzed, for which the energy radius deviation is 11 μm at 90% encircled energy. The experimental results demonstrates the potential of the APPJ approach for the manufacturing of complex surface topographies.

## 1. Introduction

High-powered laser systems require precise control of the laser beam shape and energy distribution in the target plane [1]. The continuous phase plate (CPP), as a beam-shaping optical element, can manipulate the incident laser to allow beam shaping and smoothing with complex surface topographies [2]. The surface topography of the CPP, having multiple spatial scales, high peak-to-valley (PV) values, large gradients, and high accuracy, causes much difficulty in fabrication [3].

Subaperture technology is mainly used to imprint the topography deterministically to obtain CPP elements [4]. Menapace et al. [5] developed the magnetorheological finishing (MRF) technique to fabricate large-aperture CPPs for the National Ignition Facility (NIF), in which the spatial periods of the surface topography are usually larger than 4 mm and the PV values are as high as 8.6 μm [6,7]. Microstructures with smaller spatial periods are difficult to process with MRF due to the limitations of tool sizes. Ion beam figuring (IBF) has the potential to process smaller period structures, as the beam sizes can be changed easily with a shielding diaphragm to as small as 1 mm. Xu et al. [8,9] used the ion beam figuring (IBF) approach with different beam diameters based on the frequency filtering method to improve the machining accuracy and efficiency of CPPs. However, the low removal rate limits its application for large-aperture CPPs.

The atmospheric pressure plasma jet (APPJ) approach is an efficient manufacturing technology with the advantages of high material removal efficiency, adjustable tool function size, and no subsurface damage. It is based on the chemical reaction removal mechanism, involving a chemically reactive plasma jet driven with radio frequency (RF) power under atmospheric pressure conditions. The plasma jet is fed by a mixture of fluorine-containing reactive gases (NF_3_, CF_4_, SF_6_). The reactive gases dissociate in the plasma, producing chemically reactive fluorine radicals that react with the workpiece surface to form volatile products. In the case of silicon-based materials, SiF_4_ is formed and exhausted as a waste product. It has been shown that plasma jet technology is a very efficient tool for the treatment of optical surfaces made of silicon, silicon-based materials such as fused silica for damage-free optics in high-powered laser systems, and silicon carbide for space applications.

The plasma jet technology combines the advantages of a non-contact machining technique with material removal rates comparable to traditional polishing methods. As a key advantage, subsurface damage is avoided owing to the material being removed by plasma-assisted chemical etching without any mechanical or physical contribution. The high-density distribution of reactive species can allow high material removal rates. Furthermore, the lateral dimensions of the plasma jets can be adjusted easily from around 14 mm down to the sub-mm range. Thus, these plasma jets are suitable for pre-shaping with high machining depths and for deterministic shape correction with high spatial resolution. Due to the pure chemical removal mechanism, the surface roughness increases while surface contaminants are efficiently removed the during processing. At present, the disadvantage of the plasma jet approach is solved by using a combined processing chain.

Several fluorine-plasma-based machining techniques have been developed for optical surface fabrication and freeform manufacturing. Castelli et al. [10,11] adopted the reactive atom plasma (RAP) approach for large-optics rapid surface figuring, bringing the figure error corrections down to 30 nm RMS on a ULE workpiece measuring 400 mm in diameter with a 3 m radius of curvature (ROC). Yamamura et al. [12,13] developed the plasma chemical vaporization machining (PCVM) approach to correct thickness deviations of quartz crystal wafer, whereby the thickness distribution for 14.4 nm PV was obtained after two correction steps. Arnold et al. [14,15,16] proposed plasm jet machining (PJM) and investigated the effects of the surface temperature on the etching rate. A three-dimensional finite element heat transfer model was built to assess the spatiotemporal variations of the surface temperature and temperature-dependent material removal. Su et al. [17] applied atmospheric pressure plasma processing (APPP) to CPP fabrication, whereby 320 mm × 320 mm CPP of B33 with 2.78 μm PV was fabricated and the RMS of the form error was 96 nm. Li [18,19] proposed a multiaperture plasma processing method to structure a 30 mm × 30 mm CPP, for which the peak-to-valley error was 163.4 nm. The potential of plasma processing for manufacturing CPP has been proven but the fabrication accuracy and efficiency need to be further improved to meet the performance requirements.

In this paper, an optimized iterative algorithm for high-accuracy and high-efficiency CPP manufacturing is presented. The APPJ system and surface topography processing are first introduced and the removal function characteristics are investigated to obtain the optimal processing parameters. Then, an optimized iterative algorithm based on the dwell point matrix and FFT is proposed to improve the accuracy and efficiency of the APPJ processing. The experimental processing is carried out to validate the accuracy and efficiency of the APPJ in fabricating CPP. Finally, the far-field distribution characteristics of the processed CPP are calculated.

## 2. Experiment

### 2.1. Experiment Setup

**Figure 1:**
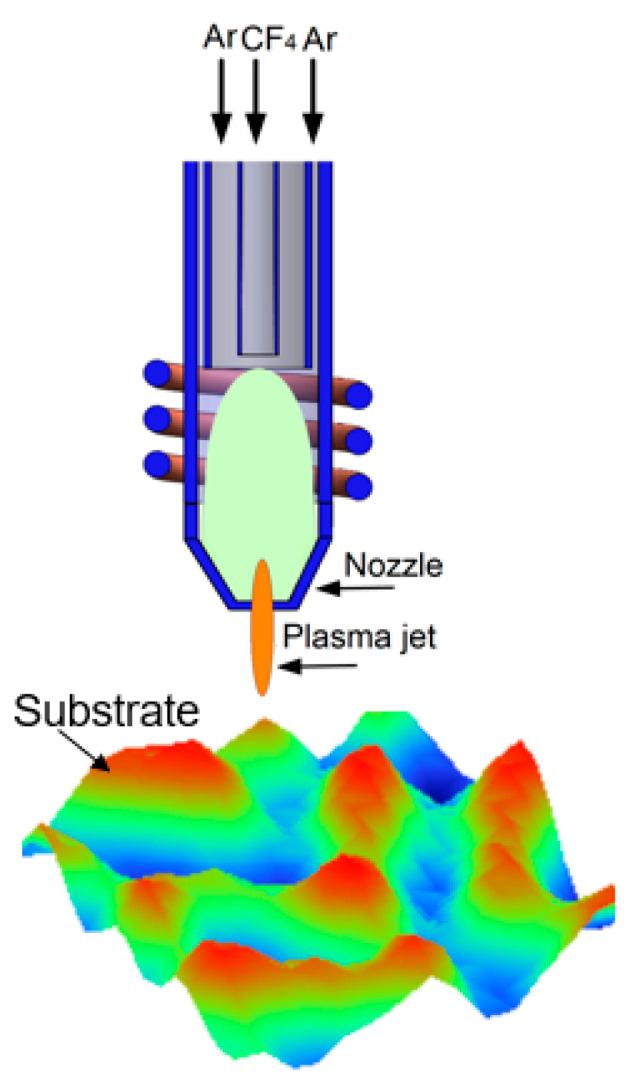
Schematic diagram of the APPJ used for CPP processing.

The APPJ made use of a radio frequency inductively coupled plasma (ICP) torch as a tool to generate the plasma jet. The plasma jet source consisted of three coaxially arranged conducting tubes guiding the plasma gas Ar and reactive gas CF_4_ together to a nozzle. The plasma jet was generated through the excitation energy (radio frequency at 13.56 MHz) and the CF_4_ was decomposed into active fluorine atoms. These reactive fluorine atoms acted as the main reactant, which was carried by plasma jet and reacted with the substrate to form the volatile reaction products SiF_4_ and CO_2_. A schematic diagram of the APPJ used for CPP processing is shown in Figure 1.

The lateral dimensions of the primary plasma discharge can be modified within a range between 2 mm and 14 mm by varying the inner diameter of the exit nozzle or by adding additional beam-shaping nozzles and apertures. In this way, the material removal rates of 0.01 to 10 mm^3^/min are achieved.

### 2.2. APPJ Processing of Surface Topography

**Figure 2:**
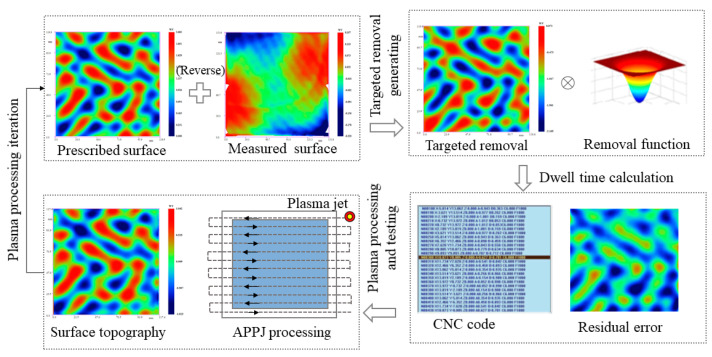
The fundamentals of APPJ processing.

The fundamentals of the APPJ processing flow chart include targeted removal, dwell time calculations, plasma processing, and testing, as shown in Figure 2. First, the prescribed surface data were inverted and superimposed with the existing measured surface figure and the targeted removal map was obtained. Then, the dwell time and residual error were calculated through the removal function and the targeted surface deconvolution iteration, while the CNC program was also generated according to the dwell time and path. After this, the APPJ processing was performed to structure the phase topography on the optic substrate. The residual error was obtained by comparing the measured surface topography with the prescribed surface. Several plasma processing iterations were required until effective convergence of the residual error was achieved.

The APPJ approach uses the removal function and dwell time to differentially remove material from areas of an optic so that the desired surface can be obtained. Two main aspects in the plasma surface topography processing deserve mention, namely the size and removal rate of the removal function used to structure the topography. The physical characteristics of the removal function determine the accuracy and success of the plasma processing on the surface topography. The dwell time calculation is another key aspect during APPJ processing, in which the APPJ process integrates interferometry and a computer algorithm to generate the required instrument stage motions to deterministically remove material surfaces. The algorithm attempts to converge to a solution that minimizes the RMS of the residual error between the prescribed surface and plasma processing surface.

## 3. Methods

### 3.1. Removal Function 

**Figure 3:**
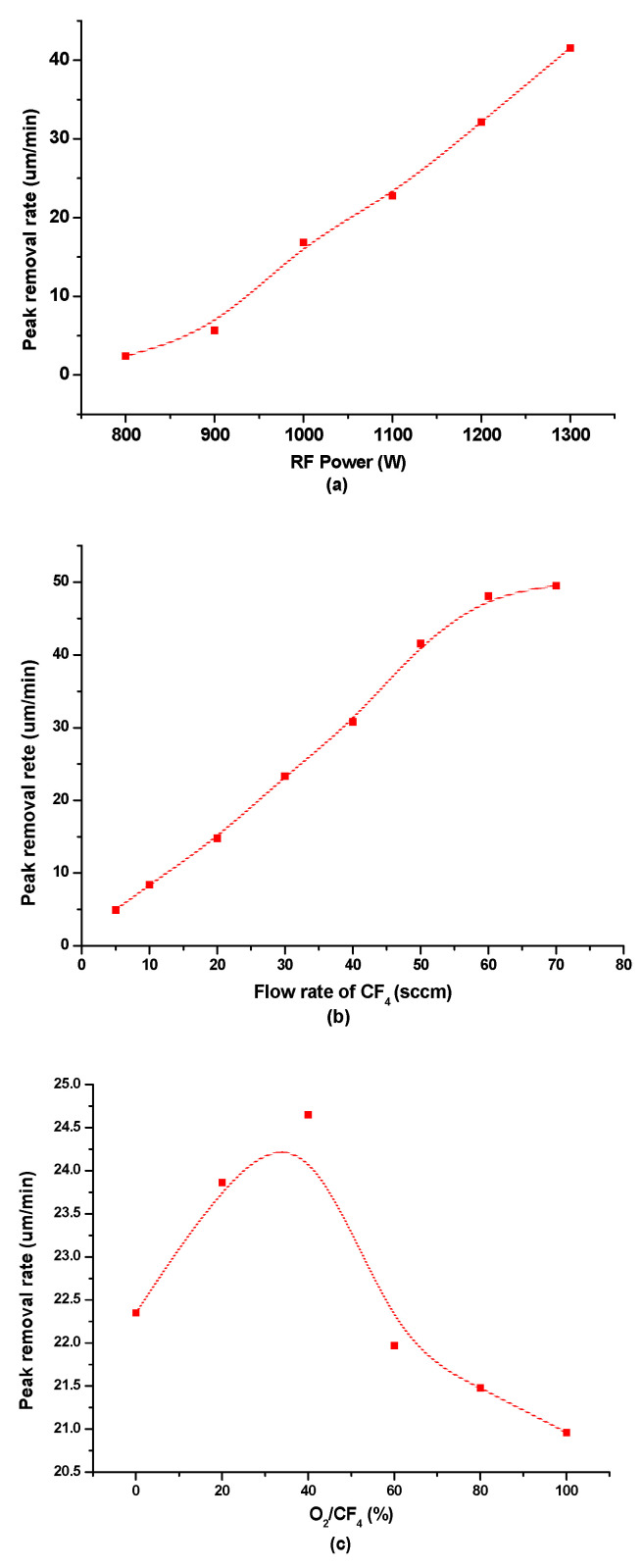

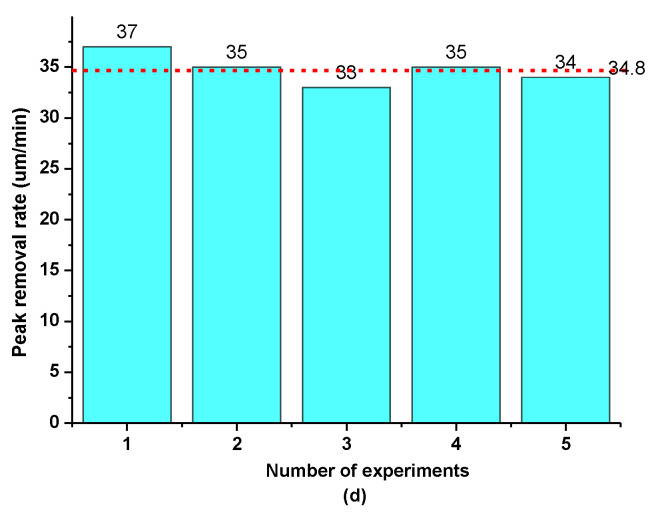
Influence on peak removal rates of plasma parameters. (**a**) RF power. (**b**) Flow rate of CF_4_. (**c**) O_2_/CF_4_ ratio. (**d**) Repeatability of the removal function.

The most important factor to achieve precise etch depths for phase plate fabrication is well-founded knowledge of the etch rate and its stability during the etching process. Therefore, the peak removal rates were investigated closely as functions of the plasma RF power and the CF_4_–Ar gas mixture flow and ratio. The key experimental parameters and variation range for removal characteristic groups are listed in Table 1. In practical plasma processing, the plasma jet stability, shape of the influence function, and material removal efficiency need to be considered. Combined with the experimental results, the processing parameters were determined. As the cooling gas, Ar is immitted through the external tube with a tangential inlet at a flow of 16 slm (standard liter per minute). The plasma gas, also Ar, is immitted through the intermediate tube at a rate of 1 slm, whereas the reactive gas CF_4_ is kept at a flow rate of 5–70 sccm (standard cubic centimeter per minute, 1 sccm = 10^−3^ slm) when entering the center tube. The effects of the plasma parameters on the peak removal rates are shown in Figure 3.

Figure 3a shows the peak removal rates with the RF power values ranging from 800 W to 1300 W. It can be seen that the peak removal rates significantly increase with the increase of RF power, which provides reactive species to promote material remove. Figure 3b shows the peak removal rates with the flow rates of the reactive gas CF_4_ ranging from 5 sccm to 70 sccm. It is clear that the peak removal rates increase linearly with the CF_4_ flow, reaching 48 μm/min at 60 sccm; when the CF_4_ flow rate exceeded 70 sccm, the plasma discharge became unstable. Figure 3c shows the peak removal rates with the O_2_/CF_4_ ratio, whereby the addition of O_2_ to CF_4_ gas leads to fluorine-rich plasma, which can improve the removal rate, while the peak removal rate reaches a peak value at about 40% of O_2_/CF_4_ and then decreases upon the addition of O_2_. Figure 3d shows the repeatability of the removal function with processing parameters at 1100 W RF power, 60 sccm CF_4_ flow, and 40% O_2_/CF_4_ ratio, where five removal functions are etched with the same parameters and the maximum deviation of the peak removal rate is about 6.3%.

### 3.2. Dwell Time Algorithm

The dwell time is the input control for surface topography structure configuration, meaning the dwell time algorithm is the key issue in APPJ processing. For a conventional dwell time calculation, the removed material *F*(*x*, *y*) is equal to the convolution of the removal function *R*(*x*, *y*) and the dwell time *T*(*x*, *y*) is given as follows:

(1) F(x,y)=R(x,y)∗T(x,y)

The dwell time *T*(*x*, *y*) can be obtained by calculating the deconvolution of the removed material *F*(*x*, *y*) and the removal function *R*(*x*, *y*) [20]. However, with this deconvolution calculation process, it is difficult to achieve convergence for the prescribed surface topography and the calculation process is also time-consuming, especially for complex structure components, for which the calculation scale is large and the accuracy and efficiency of conventional calculations are difficult to match with the processing requirements. Therefore, an iterative algorithm based on the dwell point matrix and fast Fourier transform (FFT) was proposed to achieve high accuracy and efficiency in the dwell time calculation.

**Figure 4:**
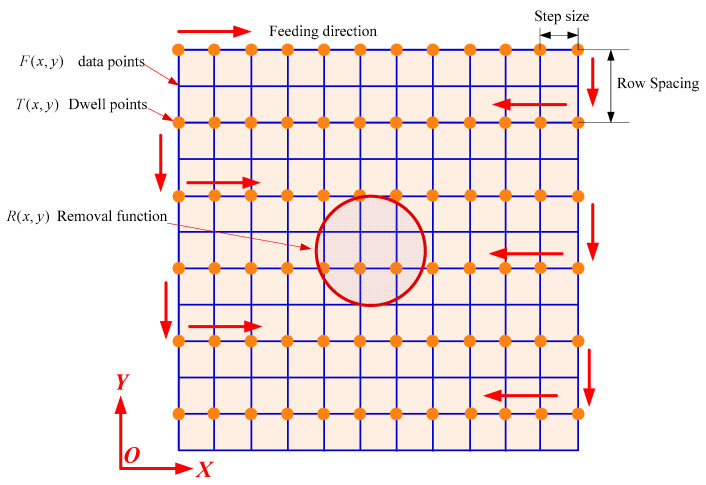
Schematic diagram of the material removal during plasma processing.

During plasma processing, the plasma jet scanning follows the raster path. A schematic of the plasma jet with the raster path is shown in Figure 4.

Assuming that the number of original surface data points is M × N and the number of dwell time points is U × V, the material removal convolution equation can be converted into the matrix-based form as follows:

(2) $$\left[\begin{array}{cccc} f_{11} & f_{12} & \cdots & f_{1N}\\ f_{21} & f_{22} & \cdots & f_{2N}\\ \vdots & \vdots & \ddots & \vdots \\ f_{M1} & f_{M2} & \cdots & f_{MN} \end{array}\right]=\left[\begin{array}{cccc} r_{11} & r_{12} & \cdots & r_{1Q}\\ r_{21} & r_{22} & \cdots & r_{2Q}\\ \vdots & \vdots & \ddots & \vdots \\ r_{P1} & r_{P2} & \cdots & r_{PQ} \end{array}\right]*\left[\begin{array}{cccc} t_{11} & t_{12} & \cdots & t_{1V}\\ t_{21} & t_{22} & \cdots & t_{2V}\\ \vdots & \vdots & \ddots & \vdots \\ t_{U1} & t_{U2} & \cdots & t_{UV} \end{array}\right]$$

Generally, the number of dwell points is less than that of the surface data points (U < M, V ≤ N) and the size of the dwell time matrix ***D*** is not equal to the original data matrix ***P***, so the dwell time matrix ***D*** and the removal function matrix ***R*** cannot be directly used for the convolution operation to obtain the matrix ***P*** [21].

To achieve fast calculation without loss of accuracy when calculating the dwell time, the concept of the dwell point matrix ***DP*** is proposed in this paper. The size of matrix ***DP*** is M × N, which is equal to the original data matrix ***P***, while the value of the dwell point is 1 and of the non-dwell point is 0. The dwell time matrix ***T*** performs the matrix Hadamard product operation [22,23] with the dwell point matrix ***DP***, as shown in Equation (3), so that the value of the non-dwell point in the dwell time matrix is equal to zero, which ensures that the amount of material removal is calculated only when the removal function is at the dwell point. The standard convolution method can be used to calculate the amount of material removal with the dwell point matrix ***DP***:

(3) $$T=\left[\begin{array}{cccc} t_{11} & t_{12} & \cdots & t_{1V}\\ t_{21} & t_{22} & \cdots & t_{2V}\\ \vdots & \vdots & \ddots & \vdots \\ t_{U1} & t_{U2} & \cdots & t_{UV} \end{array}\right]•\left[\begin{array}{cccc} 1 & 1 & \cdots & 1\\ 0 & 0 & \cdots & 0\\ 1 & 1 & \cdots & 1\\ \vdots & \vdots & \ddots & \vdots \\ 0_{M1} & 0_{M2} & \cdots & 0_{MN} \end{array}\right]=\left[\begin{array}{cccc} t_{11} & t_{12} & \cdots & t_{1N}\\ t_{21} & t_{22} & \cdots & t_{2N}\\ \vdots & \vdots & \ddots & \vdots \\ t_{M1} & t_{M2} & \cdots & t_{MN} \end{array}\right]$$

When the calculation scale is large, the calculation speed of the matrix convolution according to the definition of the convolution is slow. To improve the speed of the convolution calculation, as the spatial domain convolution calculation is equal to the frequency domain product operation, in this paper we propose using a fast Fourier transform (FFT) algorithm for convolution calculations.

(4) T∗R=IFFT(FFT(T)•FFT(R))

In Equation (4), FFT represents the fast Fourier transform and IFFT represents the inverse fast Fourier transform. In the convolution calculation, first we perform the FFT transformation on the matrix and then separately perform the Hadamard product operation on the results of the FFT transformation. Finally, we perform the inverse FFT on the multiplied result to obtain the final convolution calculation result. The convolution calculation based on the FFT has a very fast calculation speed compared to the convolution calculation according to the definition in the large-scale calculation.

**Figure 5:**
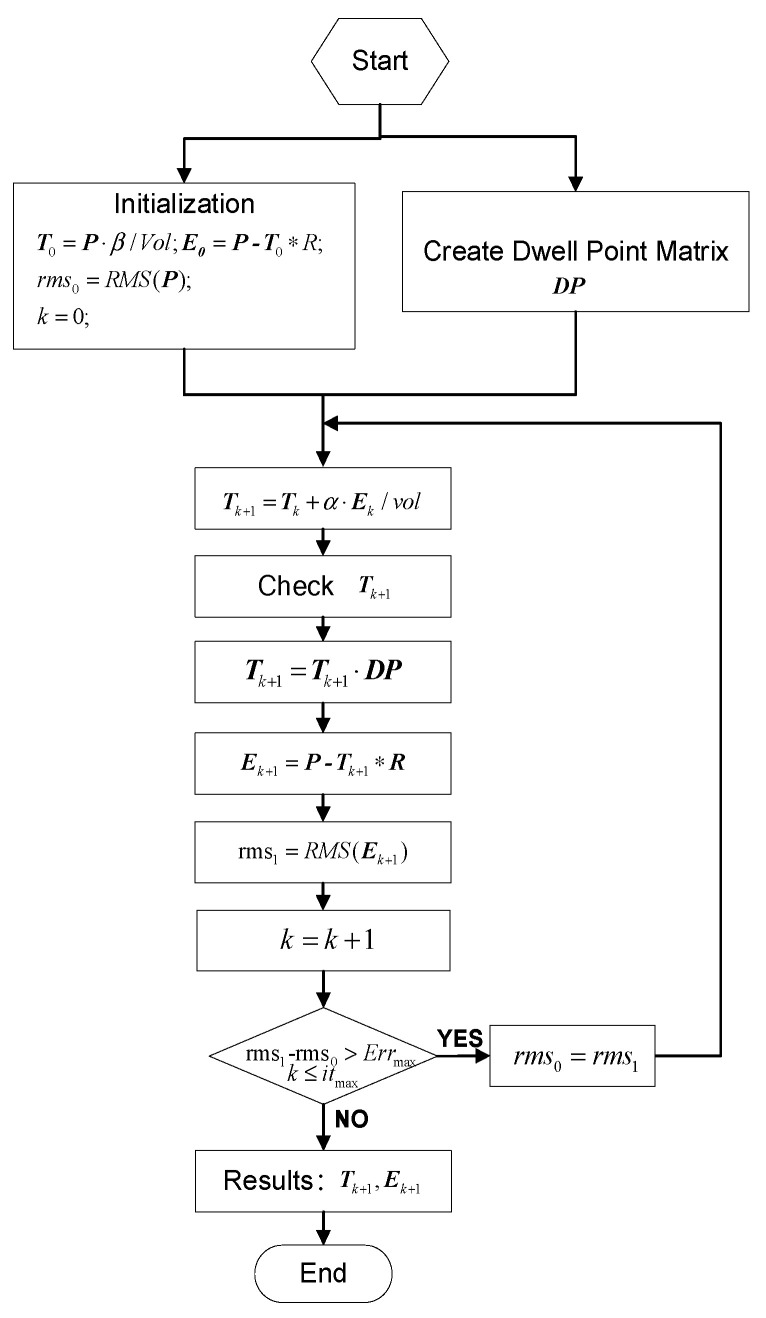
Flow chart of the dwell time algorithm.

A flow chart of the dwell time algorithm is shown in Figure 5. The dwell point matrix ***DP*** is established according to various parameters, such as the line spacing and step length. Once the necessary parameters have been chosen, the initial value of the dwell time, the permission iteration error Err_max_, and the maximum iteration number it_max_ are set. Then, the dwell time matrix T is calculated with the main iterative loop, then the dwell time matrix T is multiplied by the Hadamard product matrix DP and the residual error matrix E is calculated using a multicore parallel FFT convolution calculation. Finally, the iterative loop is terminated when the calculated residual error is less than the permission error and the dwell time $T_{k+1}$ and residual error $E_{k+1}$ can be obtained.

## 4. Results

**Figure 6:**
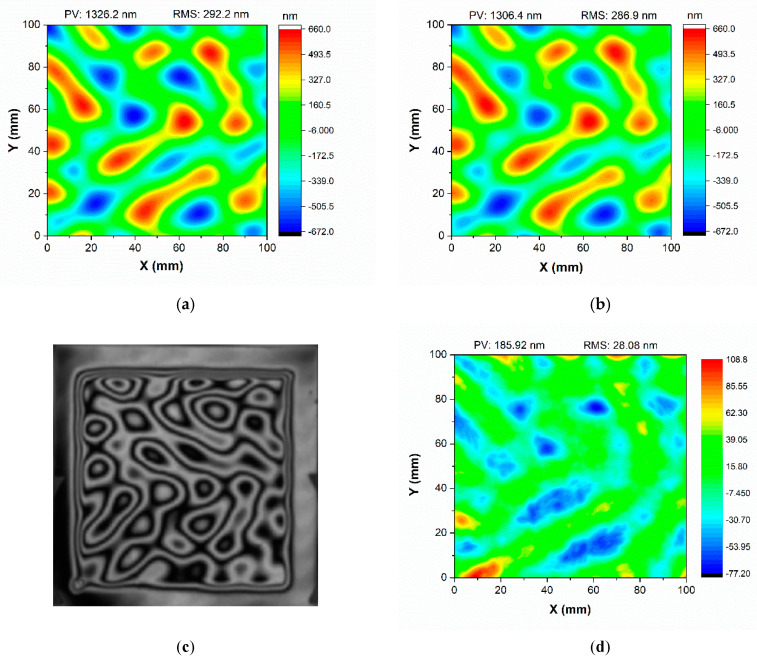
Machining example of a CPP substrate: (**a**) prescribed surface: PV = 1326.2 nm, RMS = 292.2 nm; (**b**) plasma-processed surface, PV = 1306. 4 nm, RMS = 286.5 nm; (**c**) interferometer measurement; (**d**) plasma-processed residual errors of the CPP.

The dwell time iterative calculation was used to fabricate a 120 mm × 120 mm × 5 mm CPP substrate. The prescribed surface of the CPP has a random surface topography with 1326.2 nm PV and 292.2 nm RMS, as shown in Figure 6a. All figuring experiments were performed in our self-developed APPJ system. After the experiment, the 4D interferometer was used to measure the CPP and the specified evaluation aperture for the CPP was 100 mm × 100 mm. After four plasma iteration steps, the surface topography measurements for the CPP were 1306.4 nm PV and 286.5 nm RMS, as shown in Figure 6b. The interferometer measurements and a photo of the substrate after the APPJ process are shown in Figure 6c. The actual residual error after the plasma process was 28.08 nm, as shown in Figure 6d.

**Figure 7:**
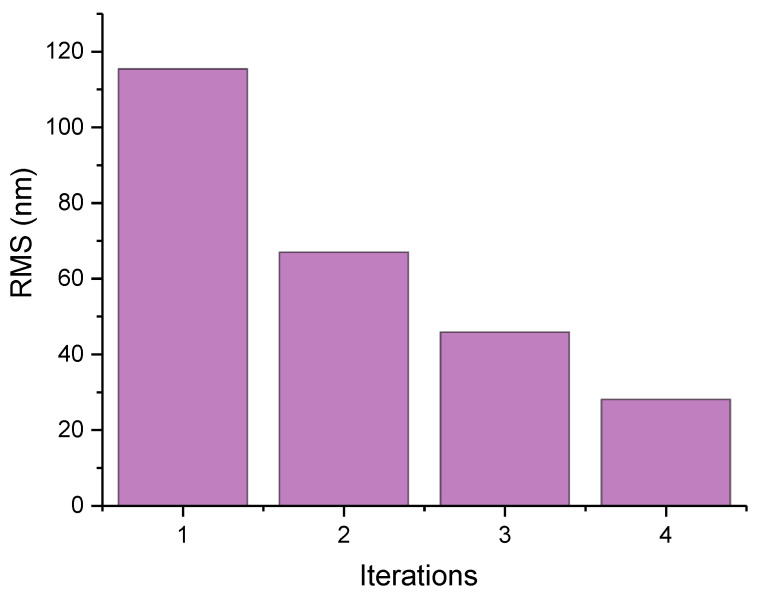
Convergence of the residual error at the four plasma processing iteration steps.

During plasma processing, further iteration steps comprise the dwell time matrix calculation using the modified topography. Several iterations are performed until the residual error obtained from the difference between the measured topography and the designed topography is less than that required by the specifications. The topographical profiles have RMS errors of <30 nm relative to the idealized CPP prescription. Figure 7 shows the convergence of the residual error of the material removal at four iteration steps for the CPP shape. After the first plasma processing stage, the surface topography was generated and a residual figure error of 115.413 nm remained, after which a further three correction steps were applied, resulting in a residual figure error of 28.08 nm.

## 5. Discussion

**Figure 8:**
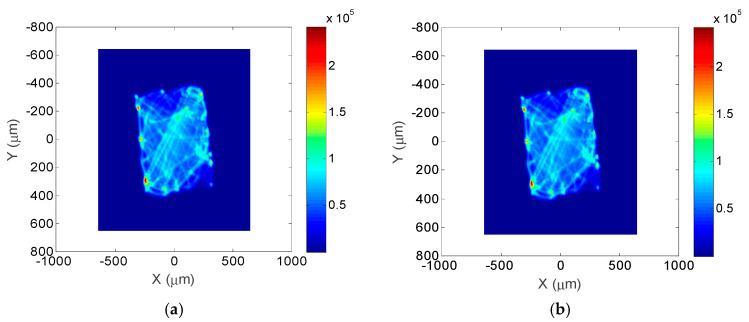
Far field distribution of the prescribed surface and plasma-processed surface: (**a**) Far-field distribution of prescribed surface; (**b**) Far-field distribution of plasma-processed surface.

The far-field characteristics of the surface shape can be analyzed using numerical simulation, whereby an ideal plane shines light through the component, making the far field of the modulated beam a direct reflection of the far-field characteristics of the CPP component, for which the wavelength λ = 633 nm and the focal distance d = 200 mm are used in the calculation. Figure 8 shows the far-field focal spot distribution of the prescribed surface and the plasma-processed surface, respectively. From the simulation results, the fabricated plasma has a reasonable beam-shaping function as the prescribed surface.

**Figure 9:**
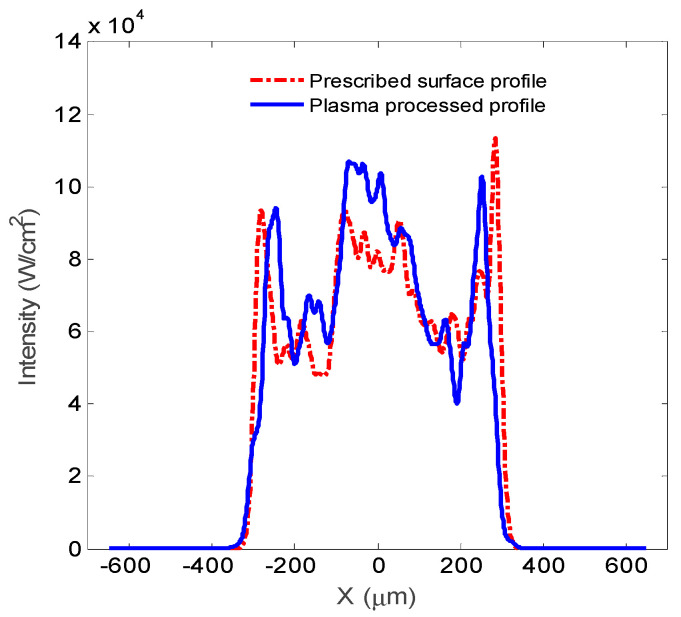
One-dimensional distribution of the focal spot.

To provide more information on the far-field distribution, the one-dimensional distributions of the far field focal spot are plotted in Figure 9. It can be seen from the figure that for the phase plate, the one-dimensional distribution of the focal spot is regular and the energy is concentrated. The cross-section profile of the plasma-processed surface is mainly consistent with prescribed surface profile, while the waveform of the intensity indicates non-uniformity of the energy distribution.

**Figure 10:**
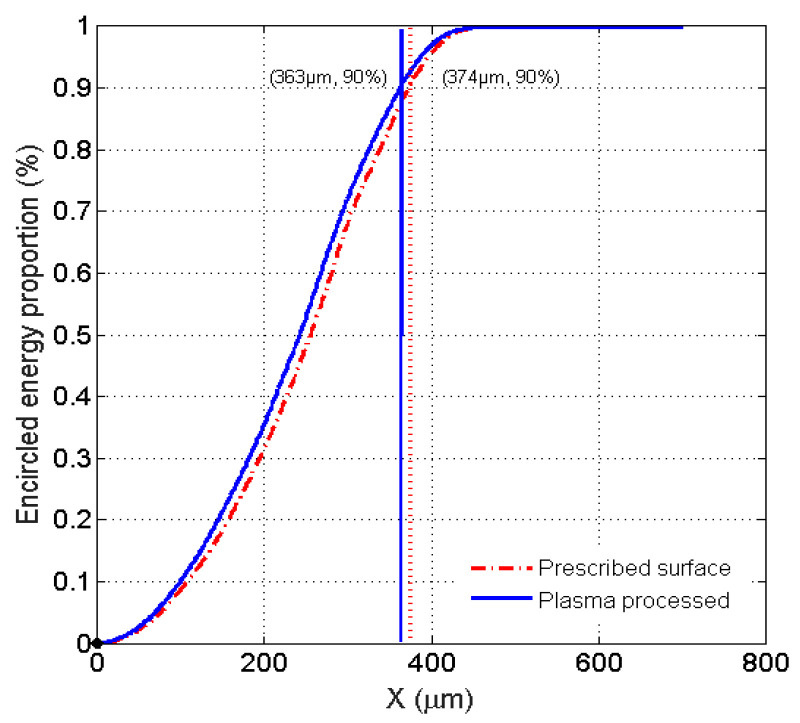
The relationship between the energy proportion and the diameter from the centroid.

The non-uniformity is scaled by the focal spot contrast in a circle with the energy included. The relationship between the energy proportion and the diameter from the centroid are given in Figure 10. The 90% encircled energy radius values for the prescribed surface and plasma-fabricated surface are 374 and 363 μm, respectively. The energy radius deviation is 11μm, which is less than the specification of 15 μm.

## 6. Conclusions

In this paper, the figuring capability of the atmospheric pressure plasma jet technology for the spatial surface topography was proven at 28.08 nm RMS. An optimized iterative algorithm based on the dwell point matrix combined with the FFT convolution calculation method was proposed in order to improve the convergence accuracy and efficiency of the dwell time calculation. The experimental results verified that this optimized method could efficiently imprint a prescribed surface topography of 120 mm × 120 mm CPP with a residual figure error of 28.08 nm (RMS) after 1.6 h of plasma processing. Comparing the far-field focal spots of the prescribed CPP and fabricated CPP, the energy radius deviation was 11μm at 90% encircled energy. Meanwhile, the peak removal rate of the APPJ system reached 48 μm/min, showing the high efficiency of the system for large-aperture optics. This study provides a new technical option for the fabrication of large-aperture optics with complex surface topographies. Ongoing process development for the APPJ process is expected to confirm its effectiveness for surface structures of wide spatial wavelength ranges and its advantages for lightweight optical components such as SiC.

## Figures and Tables

**Table 1:** Experimental parameters used for investigation of the removal characteristics.

Parameter	Value
Ar flow rate	16 slm
RF power	800~1300 W
CF_4_ flow rate	5~70 sccm
Ratio of O_2_ and CF_4_	0~100%
Processing distance	12 mm
Dwell time	3 s
